# Identifying Biomarkers Using a Portable, Home-Based Eye-Tracking System to Predict Short-Term Visual Fatigue Deterioration: Prospective Observational Feasibility Study

**DOI:** 10.2196/84479

**Published:** 2026-04-28

**Authors:** Fan Song, Guangyu Li, Jian Zhang, Zhen Tian, Mingge Li, Mengying Lai, Mingguang He, Yanxian Chen

**Affiliations:** 1 School of Optometry Hong Kong Polytechnic University Hong Kong China (Hong Kong); 2 Research Centre for SHARP Vision Hong Kong Polytechnic University Hong Kong China (Hong Kong); 3 Lab of Brain-inspired Algorithm and Model Guangdong Institute of Intelligence Science and Technology Zhuhai, Guangdong China; 4 State Key Laboratory of Ophthalmology Zhongshan Ophthalmic Center Sun Yat-sen University Guangzhou, Guangdong China; 5 Department of Ophthalmology Shenzhen Nanshan People’s Hospital Shenzhen, Guangdong China; 6 The Center for Eye and Vision Research Hong Kong China

**Keywords:** eye tracker, asthenopia, blink, eye movement, prediction, home monitoring, pupil dynamics, visual task

## Abstract

**Background:**

The escalating prevalence of screen-related eye fatigue has become a health burden in the digital era worldwide, yet routine monitoring relies largely on subjective reports. This underscores the urgent need for clinically applicable, objective diagnostic solutions. Ocular metrics provide an objective method to assess computer vision syndrome, or asthenopia.

**Objective:**

This study aimed to develop and evaluate an integrated at-home system for predicting short-term deteriorated asthenopia using objective ocular metrics. This system classifies the short-term risk level for practical monitoring and automatically generates a session report that summarizes metrics to complement symptom-based evaluation.

**Methods:**

We developed EyeFatigue Tracker, an integrated at-home system delivered via a desktop app, comprising a head-mounted device to record binocular infrared eye videos, a deep learning (DL) model to extract ocular metrics, and a machine learning (ML) classifier to estimate asthenopia risk. The DL model, trained on an in-house dataset, segments the palpebral fissure, pupil, and iris from recorded videos to derive ocular metrics. To build the prediction model, participants were recruited to complete a 1-hour computer gameplay session. Changes in the Computer Vision Syndrome Questionnaire (CVS-Q) scores served as the primary outcome measure to classify participants into deteriorated and nondeteriorated asthenopia groups. Metrics showing significant between-group differences were used as inputs for four ML models, including support vector machine (SVM), decision tree, extreme gradient boosting (XGBoost), and random forest, to identify deteriorated asthenopia. Model performance was evaluated with fivefold cross-validation.

**Results:**

This study enrolled 38 participants aged 19-31 (mean 24.8, SD 3.11) years. Following visual tasks, participants’ CVS-Q scores were higher compared to baseline values (mean 9.21, SD 4.57, vs mean 6.76, SD 3.76; *P*<.001). Alongside the critical flicker fusion frequency (CFF), nine key features were selected as predictive indicators, with the top five reflecting fissure length variability (variance, coefficient of variation, and SD), average blink duration, and pupil size variability (coefficient of variation). Most ML models exhibited high discriminative ability, with the random forest achieving the best overall performance (mean accuracy 0.720, SD 0.035; mean area under the receiver operating characteristic curve 0.850, 95% CI 0.830-0.860).

**Conclusions:**

The findings highlight the potential of objective indicators in identifying individuals at risk for asthenopia following computer gameplay. The ML models using ocular biomarkers identified in this study achieved plausible discriminative ability in detecting deteriorated asthenopia. EyeFatigue Tracker functions as an integrated, at-home system that produces a risk level prediction and a concise session report, supporting early detection and informing preventive care in real-world settings.

## Introduction

### Background

With advancements in technology and the widespread use of digital devices, complaints about asthenopia, or computer vision syndrome, have rapidly increased [1]. The worldwide prevalence of asthenopia has reached 60 million, with an additional 1 million new cases reported annually [2]. Asthenopia encompasses a range of subjective symptoms, including eye fatigue, eye swelling, dryness, blurred vision, and orbital pain [3,4], often along with musculoskeletal discomfort, such as neck pain [5]; neuropsychological symptoms, such as headaches and dizziness [6]; and general malaise [7]. Although typically transient, the symptoms can be recurrent and persistent [8]. These symptoms affect not only visual comfort but also overall health and work productivity [2]. The increasing prevalence of visual fatigue has thus become a significant burden on both individuals and society, emphasizing the urgent need for effective diagnostic and management strategies.

### Existing Assessment Methods and Research Gap

Despite its growing impact, there is no unified standard for diagnosing asthenopia. Existing assessment approaches can broadly be categorized into subjective symptom questionnaires and objective physiological or ocular video–based methods. Most studies rely on subjective questionnaires, with the Computer Vision Syndrome Questionnaire (CVS-Q) being a widely used tool for assessing the frequency and severity of asthenopia symptoms [3]. Such questionnaires are free to use in large populations, but participants still need to complete multiple items and calculate total scores. In routine or remote use, they may also have questions about specific items that cannot be answered promptly by eye care professionals, which makes these tools less practical for frequent at-home monitoring. In addition, objective physiological measures, such as the critical flicker fusion frequency (CFF), have been explored [9,10]. Although the CFF offers a quantifiable approach, its reliability in assessing asthenopia and its potential as a surrogate measure remain controversial [11]. Other objective metrics, such as blinking patterns, eye movement metrics, accommodative function, and pupil dynamics, offer additional quantitative insights into asthenopia [12-15]. These physiological metrics can provide reproducible endpoints but usually require dedicated clinical equipment and in-clinic testing, which reduces their feasibility for routine, at-home monitoring. For example, accommodative function reflects the eye’s ability to focus but requires specialized equipment and clinical expertise, limiting its practicality. Similarly, eye-tracking devices, such as the Tobii system, can capture eye movement data, including fixation and saccade metrics [16]. However, these devices are often costly and require sophisticated analysis to interpret the data accurately [17]. More recently, some groups have combined blink, pupil, and eye movement features into portable platforms, such as smartphone-based eye movement–recording apps and virtual reality (VR) headsets with integrated eye tracking, to assess visual behavior and visual fatigue in everyday digital tasks [18,19]. Beyond these approaches, video-based deep learning (DL) methods have been used to recognize ocular anatomy in other domains, such as cataract surgery and biometrics [20-22]. Previous studies have used explicit image feature extraction steps to improve pupil and iris recognition performance in cataract surgery videos or eye gaze–tracking tasks [23-25]. Clinical studies have also applied objective pupillometry to quantify how cataract surgery affects pupil size and constriction dynamics [26]. Overall, these systems show that it is possible to automatically extract eye structure information from videos in controlled, task-specific settings, but they were not developed to monitor visual fatigue or short-term changes in symptoms during everyday screen use. In the context of visual fatigue, DL methods have been used to extract blinking patterns (eg, frequency and duration) and pupil dynamics (eg, accommodation speed) from video data to assess visual fatigue [14,27]. Although these approaches show promise, no system has yet succeeded in integrating these multiple objective metrics into a portable, convenient, and cost-effective solution for the comprehensive evaluation of asthenopia.

### Objectives and Contributions of This Study

To address these challenges, we proposed a novel portable, at-home system that uses DL algorithms to simultaneously measure blinking patterns, eye movement metrics, and pupil parameters. By integrating these metrics, the device may provide a more comprehensive and objective assessment of asthenopia. Specifically, this study (1) developed an integrated at-home system that records binocular infrared (IR) eye videos and derives anatomy-aware ocular metrics, (2) identified ocular biomarkers associated with short-term deteriorated asthenopia using a standardized computer gameplay protocol, and (3) evaluated multiple machine learning (ML) models to classify at-risk individuals based on these biomarkers. This approach aims to offer a practical and accessible tool for identifying individuals prone to visual fatigue in real-world settings.

## Methods

### Ethical Considerations

Ethical approval was obtained before the study began from the Institutional Review Board (IRB) of the Hong Kong Polytechnic University (reference #HSEARS20240425002). The study adhered to the tenets of the Declaration of Helsinki. Written informed consent was obtained from all participants prior to study participation, and participants were informed that they could withdraw from the study at any time without a penalty. All study data were deidentified before analysis and were stored securely to protect participant privacy and confidentiality. Participants received HK $350 (~US $45) as compensation for their participation. Potentially identifiable participant images were included only where scientifically necessary, and separate written consent for publication was obtained from the individuals concerned.

### Data Collection

This prospective observational study was conducted between July and August 2024. Participants aged 16-35 years were included. Exclusion criteria encompassed individuals with the presence of diseases that may cause eye pain or headaches, such as glaucoma, keratitis, strabismus, conjunctivitis, iridocyclitis, ocular trauma, or other self-reported conditions; color vision deficiency; illiteracy; or cognitive dysfunction.

Sociodemographic information and general health history were collected from the participants. Following this, each participant engaged in four consecutive 15-minute computer games on a laptop (2560×1600 resolution, 240 Hz refresh rate) for a total of 60 minutes of continuous screen use. The games consisted of simple but engaging tasks (maze navigation [28], block stacking [29], obstacle avoidance [30], and sports-themed minigames [31]) to maintain attention, while providing sustained visual load (see Figure S1 in Multimedia Appendix 1). The total duration and task design were chosen to create short-term, measurable changes in visual fatigue, while preserving compliance, in line with prior work showing that approximately 1 hour of continuous digital device use can significantly aggravate visual fatigue [32]. Each participant first completed a 1-minute text-reading task (Multimedia Appendix 2), then performed the four game sessions, and immediately completed the same 1-minute text-reading task. During each text-reading task, the participant wore the EyeFatigue Tracker device, which recorded their ocular videos. The task consisted of three consecutive 20-second segments with progressively reduced contrast, smaller font size, and more complex text, increasing in difficulty from easy to moderate to difficult in a fixed order, so that we could examine how ocular metrics changed as visual and cognitive load increased within a brief standardized trial. This stepwise change in contrast, font size, and text complexity is consistent with prior work showing that smaller text, lower contrast, and more demanding reading conditions can increase visual fatigue and mental workload in digital reading tasks [33,34]. For analysis, these three segments approximately corresponded to the frame ranges 200-600 (easy), 600-1200 (moderate), and 1200-1800 (difficult) after discarding the first 200 frames.

**Figure 1:**
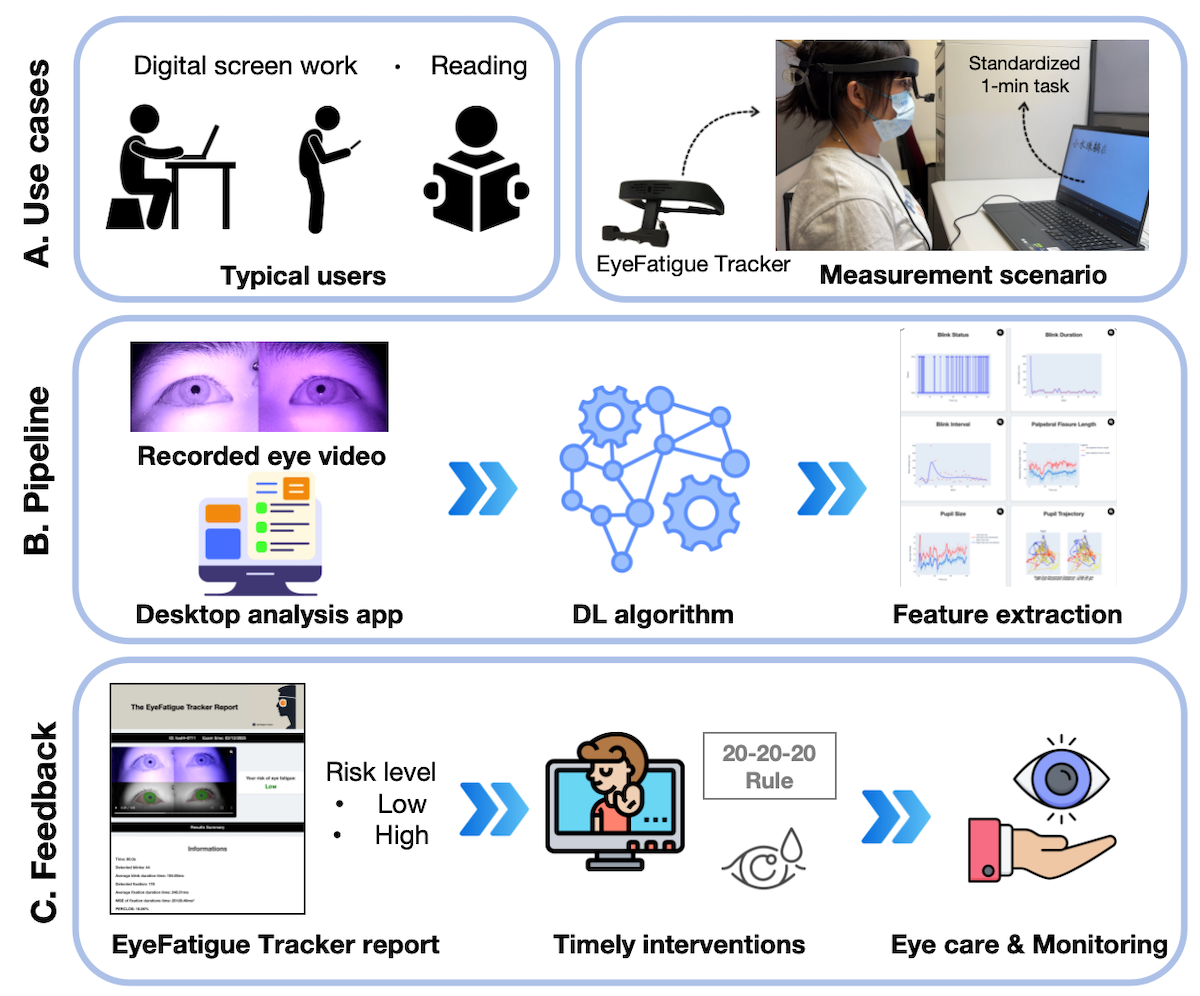
Overall flowchart of the EyeFatigue Tracker system and study workflow. (A) Typical users and standardized measurement scenario during a 1-minute reading task with the head-mounted EyeFatigue Tracker. (B) Analysis pipeline: Infrared eye videos are processed by a mask R-CNN–based segmentation model to obtain masks of the palpebral fissure, iris, and pupil. From these masks, objective ocular metrics are extracted, including blink parameters, pupil dynamics, and eye movement metrics. Statistically significant metrics are then used as inputs to ML classifiers (SVM, decision tree, random forest, and XGBoost) to estimate the short-term risk for deteriorated asthenopia (low vs high). (C) Feedback module: The desktop app displays the predicted risk level, provides a brief session report summarizing key ocular metrics, and can be used for home-based eye care, longitudinal self-monitoring, and telemedicine follow-up. DL: deep learning; ML: machine learning; mask R-CNN: mask region-based convolutional neural network; SVM: support vector machine; XGBoost: extreme gradient boosting.

Data collected included habitual visual acuity (HVA), tear film break-up time (TBUT), CVS-Q scores [3], Stanford Sleepiness Scale (SSS) scores [35], Karolinska Sleepiness Scale (KSS) scores [36], Fatigue Assessment Scale (FAS) scores [37], CFF values, and ocular metrics (blinking patterns, eye movement metrics, and pupil parameters). The CVS-Q served as the primary subjective measure of asthenopia and was used to define deteriorated and nondeteriorated asthenopia groups. The SSS and the KSS captured current and recent sleepiness, and the FAS assessed overall fatigue, allowing us to examine whether changes in ocular metrics are specifically related to visual fatigue rather than general tiredness. The CFF was included as a classical psychophysical index of visual fatigue to compare with the proposed ocular video–based metrics. The HVA was measured by the Early Treatment Diabetic Retinopathy Study (ETDRS) chart at 4 m. The TBUT was recorded as the time interval between the last blink and the first tear film dry spot following fluorescein instillation, using a slit lamp. The severity of asthenopia, current sleepiness, recent (last 10 minutes) sleepiness, and overall fatigue were assessed using the CVS-Q, SSS, KSS, and FAS questionnaires, respectively, with higher scores indicating greater severity. CFF values were measured using Handy Flicker HF-III (Neitz Instrument), and the average value of ascending and descending thresholds were recorded. A decrease in CFF values reflects increased visual fatigue. Ocular objective metrics were extracted from the 1-minute video recorded by the EyeFatigue Tracker device. The overall experimental setting and visual task timeline are illustrated in Figure 1A (measurement scenario).

### Proposed EyeFatigue Tracker System

#### System Overview

Figure 1 presents the EyeFatigue Tracker workflow used to estimate the risk of asthenopia deterioration. The EyeFatigue Tracker system classifies each 1-minute recording into a risk level (low/high) for asthenopia deterioration during at-home use. The device consists of a lightweight head-mounted binocular IR camera module, paired with a desktop analysis app, mounted on an adjustable plastic frame, which can be worn over habitual spectacles at a normal working distance from a computer screen. It connects to a standard laptop via a single USB cable and does not require a chinrest or dedicated laboratory setup, making it feasible for use in home and office environments. The hardware was assembled from off-the-shelf components to keep material costs low. Two IR cameras were used to capture videos of both eyes at a frame rate of 30 frames per second (fps). We used 940 nm near-infrared (NIR) illumination, together with an 850 nm long-pass IR filter (cut-on at 850 nm) mounted in front of the camera lens to suppress visible light from the display/ambient illumination and reduce glare-related artifacts. This rate was chosen as a practical trade-off between temporal resolution and computational cost: Blinks are brief (about 100-150 ms), so markedly lower frame rates (eg, 10-15 fps) may miss the full closing and reopening phases and distort blink duration, blink interval, and percentage of eyelid closure (PERCLOS), whereas much higher frame rates would substantially increase data size and processing time without a clear benefit for our target task. Participants wore EyeFatigue Tracker while performing a 1-minute text-reading task, during which a video containing 1800 frames (1 minute) was recorded for feature extraction. IR ocular videos were recorded at 3840×1080 pixels and 30 fps, and frames were downsampled to 480×135 pixels for training and 640×180 pixels for inference in the DL models.

#### Deep Learning–Based Eye Anatomy Recognition

We used two nonoverlapping datasets. Dataset A is an in-house eye video dataset from 100 subjects with different eye states (open, half-open, closed, left-right scanning), and we selected 1000 labeled eye images and used them exclusively to train and validate the segmentation model. Dataset B is an independent cohort recruited in this study (see the *Data Collection* section) and was used for feature extraction and predictive modeling.

Instance segmentation of ocular anatomy was implemented in Detectron2 [38] with a mask region-based convolutional neural network (mask R-CNN; residual network [ResNet]-50 backbone + feature pyramid network [FPN]), which we selected after a preliminary comparison with a U-Net–based model showed that the mask R-CNN produced more stable and accurate masks for small ocular structures (pupil boundary and palpebral fissure), while remaining feasible to train and run on a standard laptop. Prior to segmentation, IR frames underwent lightweight preprocessing to suppress overexposure and normalize intensity. Annotations were stored in Common Objects in Context (COCO)–style format and registered as Detectron2-compatible datasets. Segmentation outputs were grouped into three anatomical structures: palpebral fissure, pupil, and iris. All recordings were visually inspected, and videos with severe artifacts or segmentation failures were excluded to ensure that subsequent analyses primarily reflected true ocular behavior. The model was initialized with weights pretrained on the COCO instance segmentation task and then fine-tuned on Dataset A only (3000 iterations, batch size=2, learning rate=2.5×10⁻^4^). Due to graphics processing unit (GPU) memory constraints and the small-scale nature of the dataset, we used a batch size of two images per iteration, which is standard for a mask R-CNN in Detectron2 and provided stable convergence for this task. Training/validation/test splits were made at the participant level to avoid subject-level leakage. This model enables precise differentiation of various eye regions. We reported the Dice similarity coefficient (Dice) and intersection over union (IoU) in two forms: macro (unweighted mean across classes) and micro (global aggregate). Per structure metrics were macroaveraged across left and right eyes, whereas the microaveraged performance was obtained by aggregating all structures and images before computing the Dice, IoU, and COCO-style average precision (AP)/average recall (AR) using the Detectron2 COCOEvaluator. One-to-one instance matching was performed using the Hungarian assignment at IoU≥0.5. We also reported COCO metrics via Detectron2’s evaluator, including the AP and AR at AP averaged from IoU 0.50 to 0.95 (AP@0.50-0.95), AP at IoU 0.50 (AP@0.50), and AR averaged from IoU 0.50 to 0.95 (AR@0.50-0.95). For clinical interpretability, metrics were also summarized after aggregating to eye (palpebral fissure), iris, and pupil.

#### Assessment Metric Computation and Risk Prediction

Based on the segmentation results, we extracted blink times, blink duration, blink interval, PERCLOS, fissure length, fixation number, fixation duration, eye movement distance, and pupil constriction speed. The average blink duration represented the mean length of each blink, while the blink interval referred to the duration between two consecutive blinks. Blink times were measured as the total number of blinks per minute. PERCLOS was defined as the proportion of time during which the eyes remained at least 80% closed. The fixation number represented the number of fixations per minute, while the average fixation duration represented the average length of each fixation. The eye movement distance was computed from the pupil centroid trajectory extracted from the pupil segmentation masks. For each frame, the pupil center was estimated, and the total movement was calculated as the cumulative Euclidean distance between consecutive centroids. Frames in which the pupil was not visible (eg, during eye closure) were automatically excluded from the calculation. The pupil constriction speed indicated the rate of pupil size change during constriction and dilation. To minimize the influence of the initial eye-opening movements in the video, the variations of ocular metrics were analyzed between frames 200 and 1800. For variability features, we calculated the SD, variance, and coefficient of variation of pupil size and fissure length within each frame range (200-600, 600-1200, 1200-1800) and across all frames (200-1800) separately for the pre- and posttask recordings. The difference in each variability metric was defined as posttask minus pretask and compared between groups. The corresponding change features were then calculated as the difference between post- and pretask summary statistics. Point-wise subtraction of raw signals was not performed.

Objective metrics were compared between the deteriorated and nondeteriorated asthenopia groups, defined using changes in CVS-Q scores. Participants were classified as having deteriorated asthenopia if their posttask CVS-Q score increased by ≥3 points compared to baseline; all others were assigned to the nondeteriorated asthenopia group. Statistically significant features were selected as inputs for the four ML models for predicting short-term deteriorated asthenopia. Statistical feature filtering (*P*<.05) was used to define candidate ocular biomarkers and was not re-estimated within each cross-validation fold; this design choice may introduce selection bias and was therefore noted as a limitation. This feature-filtering step was performed on the full dataset and served to define a fixed set of input features for subsequent ML analyses, rather than as a data-driven feature selection step within the model training pipeline. These ML models include both classical classifiers (SVM and decision tree) and modern tree-based ensemble methods (random forest and XGBoost), which have shown strong and robust performance in time-series prediction tasks in previous studies [39-41]. To improve robustness and mitigate variability due to dataset partitioning, an 80/20 training-test split was first applied, with the test set fully held out from all training procedures. Model training and evaluation were then conducted on the training set using stratified fivefold cross-validation, and performance was assessed using the area under the receiver operating characteristic curve (AUROC). ML models were trained on statistically significant features (*P*<.05) after z-score normalization, with synthetic minority oversampling technique (SMOTE) oversampling applied exclusively within the training data of each cross-validation fold. Before oversampling, the dataset contained 42 fatigued and 34 nonfatigued samples; after SMOTE, the training data in each fold were balanced to 42 samples per class, while validation and test sets retained the original class distribution. Hyperparameters for the SVM, decision tree, random forest, and XGBoost were optimized using stratified fivefold grid search cross-validation on the training data. Hyperparameter tuning and cross-validation–based performance estimation were performed on the training set, while an independent test set was used only for final evaluation. Details of the model-specific evaluation parameters are provided in Table S1 in Multimedia Appendix 1. The final SVM used a radial basis function (RBF) kernel with principal component analysis (PCA) retaining 95% variance. Tree-based models (decision tree, random forest, XGBoost) were trained using their best-performing parameter combinations identified through the grid search. Performance metrics were derived from stratified fivefold cross-validation on the training set, while the independent test set was used only for an additional one-time validation of the selected models. After obtaining the AUROC for each fold, we computed the mean AUROC across all five folds to provide a more stable and generalized estimate of the model’s discriminatory ability. Feature importance was assessed using the model with the best performance, where the relative contribution of each feature to predicting deteriorated asthenopia was quantified based on its importance scores. Finally, we integrated these functions into a desktop app: users can complete a 1-miute visual task and use the app to assess their risk level. The graphical user interface is designed for nonexpert users and provides fully automated processing with no need for manual parameter tuning, which supports convenient at-home use. All data processing, feature extraction, and model training were performed in Python using Detectron2 for segmentation and scikit-learn for ML analyses. The analysis pipeline ran on a Lenovo ThinkPad laptop (Intel i9-14900HX, 32 GB RAM, NVIDIA GeForce RTX 4060 Laptop GPU), achieving an end-to-end processing time of ~4 minutes per session, while segmentation models were trained on an NVIDIA A100 GPU server.

### Data Analysis

The primary outcome measure was the presence of deteriorated asthenopia, identified by an increase of ≥3 points in the CVS-Q score following visual tasks. The sample size was calculated using G*Power 3.1 software [42], targeting a statistical power of 0.90 and a significance level (α) of .05, with an expected effect size (Cohen f=0.25). To account for a potential 20% dropout rate, the final sample size was adjusted to 38 participants. Data were presented as mean (SD) or mean (95% CI) for continuous variables with a normal distribution or as median (IQR) for nonnormally distributed variables. The Shapiro-Wilk test was used to assess data normality [43]. Paired *t* tests were used for normally distributed data [44]. Nonparametric tests, including the Mann-Whitney U test, McNemar’s test, and Wilcoxon signed-rank test, were used to handle nonnormal or ordinal data [45]. The Wilcoxon signed-rank and McNemar’s tests were used to measure within-participant changes in visual fatigue metrics over time points. The Mann-Whitney U test compared demographic characteristics, baseline characteristics, and changes in extracted objective metrics in subgroup analyses. All tests were two-tailed, with α=.05 considered statistically significant. The analysis was performed using Stata18.0 (StataCorp) [46].

## Results

### Participant Details

A total of 38 participants completed the study. Their ages ranged from 19 to 31 (mean 24.89, SD 3.11) years, and 19 (50%) were female. The mean TBUT was slightly shorter in the right eye than in the left eye (mean 6.48, SD 2.90, seconds vs mean 7.18, SD 2.72, seconds). The mean spherical equivalent was −2.23 (SD 2.30) diopters in the right eye and −1.93 (SD 2.32) diopters in the left eye. More than half of the participants (n=58%) had baseline asthenopia (CVS-Q score≥6). Participants reported a mean daily screen exposure of 9.45 (SD 3.37) hours. Their detailed baseline characteristics are summarized in Table 1.

**Table 1:** Baseline characteristics of participants (N=38).

Characteristics		Value
**Age (years)**		
	Mean (SD)	24.89 (3.11)
	Median (IQR)	26 (19-31)
Females, n (%)		19 (50)
**TBUT^a^ (seconds), mean (SD)**		
	Right eye	6.48 (2.90)
	Left eye	7.18 (2.72)
**Spherical equivalent (diopters), mean (SD)**		
	Right eye	–2.23 (2.30)
	Left eye	–1.93 (2.32)
Asthenopia^b^, n (%)		22 (58)
Screen exposure (hours/day), mean (SD)		9.45 (3.37)

^a^TBUT: tear film break-up time.

^b^Asthenopia was determined by a Computer Vision Syndrome Questionnaire (CVS-Q) score of ≥6.

### Segmentation Performance of the EyeFatigue Tracker System

**Figure 2:**
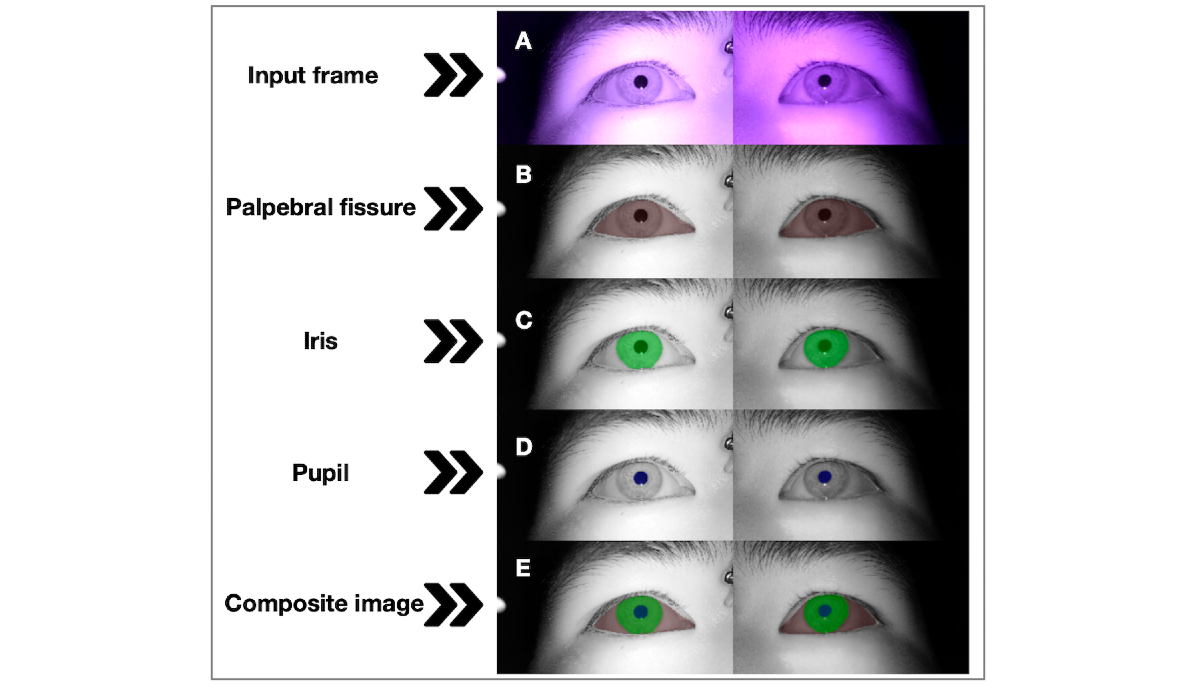
Sample outputs of the segmentation system. (A) Input frame, (B) palpebral fissure mask (red), (C) iris mask (green), (D) pupil mask (blue), and (E) final composite overlay of all masks.

The model achieved an overall mean Dice of 93.3 (SD 0.4, 95% CI 92.9-93.7) and an overall mean IoU of 87.4 (SD 0.8, 95% CI 86.7-88.1). Among individual structures, the pupil showed the highest overlap, with a Dice of 94.0 (95% CI 93.7-94.4) and an IoU of 88.8 (95% CI 88.2-89.5), followed by the iris with a Dice of 86.6 (95% CI 85.1-88.0). The palpebral fissure was the most challenging, with a Dice of 75.0 (95% CI 71.4-78.6) and an IoU of 60.2 (95% CI 55.5-64.9). COCO metrics were strong at the overall level, with a mean AP@0.50 of 99.3 (SD 0.4), a mean AP@0.50-0.95 of 80.6 (SD 1.1), and a mean AR@0.50-0.95 of 84.7 (SD 1.0). At the structure level, the iris yielded the highest AP@0.50-0.95 of 87.4, and all structures maintained an AR@0.50-0.95 of >80%. The 95% CIs were narrow across metrics, indicating stable performance across folds. This pattern is consistent with the image characteristics: the pupil has a regular circular shape and strong contrast against the surrounding iris in IR videos, whereas the iris boundary and especially the palpebral fissure are less well defined, frequently affected by eyelashes and partial eyelid closure, and therefore inherently more difficult to delineate. Table 2 summarizes all objective metrics, and Figure 2 shows representative overlays for each structure and the final composite. Additional examples of segmentation performance under different eye-closure conditions are provided in Figure S2 in Multimedia Appendix 1.

**Table 2:** Segmentation performance by structure^a^.

Structure	Dice^b^, mean (SD)	IoU^c^, mean (SD)	AP^d^@0.50, mean (SD)	AP@0.50-0.95, mean (SD)	AR^e^@0.50-0.95, mean (SD)
Pupil	94.0 (0.4)	88.8 (0.8)	99.2 (0.5)	75.9 (1.5)	82.1 (1.4)
Iris	86.6 (1.7)	76.4 (2.6)	99.7 (0.3)	87.4 (0.8)	90.7 (0.6)
Palpebral fissure	75.0 (4.1)	60.2 (5.3)	99.2 (1.0)	78.3 (1.3)	81.2 (1.4)
Overall^f^	93.3 (0.4)	87.4 (0.8)	99.3 (0.4)	80.6 (1.1)	84.7 (1.0)

^a^Per structure values are macroaveraged across left and right eyes.

^b^Dice: Dice similarity coefficient.

^c^IoU: intersection over union.

^d^AP: average precision.

^e^AR: average recall.

^f^Microaveraging across all classes.

### Comparison of Differences in Objective Metrics Before and After Visual Tasks

**Figure 3:**
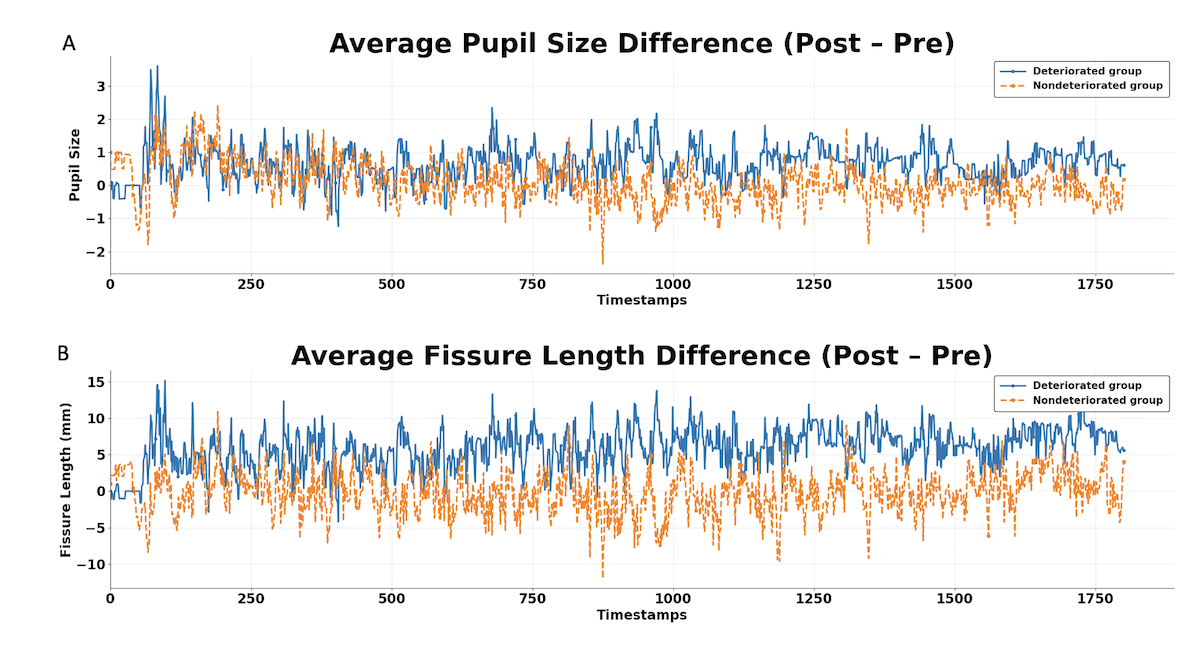
Changes in palpebral fissure length and pupil size before and after visual tasks. The x axis represents 1800 fpsframes per minute, while the y axis shows the average difference in pupil size values (A) and fissure length (B) before and after visual tasks. The blue line represents participants with deteriorated asthenopia, and the orange line represents participants without deteriorated asthenopia. Values below 0 indicate that the posttask value is smaller than the pretask value. For each time point, values were first computed per participant and averaged within each group for the pre- and posttask recordings. The posttask-pretask difference was then calculated as the difference between the two group-averaged trajectories. These curves are provided for visualization only and were not used for statistical feature extraction or ML model input. fps: frames per minute; ML: machine learning.

In addition to objective ocular metrics, subjective CVS-Q scores also showed significant worsening after visual tasks. Specifically, participants’ CVS-Q scores were higher after visual tasks compared to baseline values (mean 9.21, SD 4.57, vs mean 6.76, SD 3.76, *P*<.001). The prevalence of participants in the higher-severity group (CVS-Q scores=13-18) increased from 3 (8%) of 38 participants at baseline to 13 (34%) participants, while those without asthenopia (CVS-Q scores<6) decreased from 16 (42%) of 38 participants to 12 (32%) participants. In addition, SSS, KSS, and FAS levels all slightly increased after visual tasks (SSS: median 1, IQR 0-2; KSS: median 1, IQR 0-2; FAS: median 1, IQR 0-4). Notably, the number of participants reporting the most common complaint, dryness, rose by 4 (95% CI –6% to 27%), while those reporting excessive blinking saw a substantial increase of 15 (95% CI 21%-58%). Together, these results show that the task led to more pronounced short-term aggravation of visual fatigue during the protocol (Table S2 in Multimedia Appendix 1). Changes in these ocular metrics (ie, blink patterns, eye movement metrics, and pupil parameters) and CFF values before and after visual tasks are presented in Table 3. All differences were calculated as post- minus pretask values, so negative numbers indicated a decrease after visual tasks. The deteriorated asthenopia group exhibited a marked reduction in the average blink duration (median –4.76, IQR –49.44 to 6.74, ms). Moreover, the nondeteriorated asthenopia group demonstrated an increase in blink times (median 4, IQR 0-8). Notably, these differences were statistically significant between the two groups. Figure 3 illustrates the changes in the average pupil size and palpebral fissure length over an 1800-frame interval before and after visual tasks. The deteriorated asthenopia group showed an increase in both parameters, while these metrics remained relatively stable in the nondeteriorated asthenopia group.

**Table 3:** Comparison of differences in blink patterns, eye movement metrics, pupil parameters, and CFF^a^ values before and after visual tasks in participants with and without deteriorated asthenopia.^b^

Parameter difference	With deteriorated asthenopia	Without deteriorated asthenopia	*P* value
Blink times (per minute), median (IQR)	0 (–3 to 1)	4 (0 to 8)	.02
Average blink duration (ms), median (IQR)	–4.76 (–49.44 to 6.74)	12.21 (0 to 33.74)	.04
PERCLOS^c^ (%), median (IQR)	–0.17 (–1.11 to 0.84)	0.56 (–0.83 to 2.78)	.10
Fixation number (per minute), median (IQR)	–5 (–14 to 10)	–7 (14 to 4)	.58
Average fixation duration (ms), median (IQR)	44.88 (–14.02 to 82.15)	–6.42 (–32.03 to 45.64)	.22
Average eye movement distance^d^ (pixels), median (IQR)	36.49 (–21.60 to 75.03)	0.90 (–57.49 to 42.24)	.22
Average pupil diameter^d^ (pixels), mean (95% CI)	0.15 (–0.15 to 0.45)	0.33 (–0.00 to 0.67)	.21
Maximum speed of pupil constriction^d^ (pixels/second), median (IQR)	0.61 (–3.65 to 9.40)	0.77 (–8.27 to 9.23)	.71
Average speed of pupil constriction^d^ (pixels/second), median (IQR)	–0.32 (–1.00 to 1.41)	0.14 (–0.96 to 0.54)	.53
CFF (Hz), mean (95% CI)	–2.97 (–3.96 to –1.98)	–2.14 (–2.96 to –1.32)	.09

^a^CFF: critical flicker fusion frequency.

^b^All values are expressed as post- minus pretask; negative values denote a reduction after visual tasks.

^c^PERCLOS: percentage of eyelid closure.

^d^Average value of both eyes.

### Variability of Objective Metrics During the Reading Task

Tables 4-6 provide an overview of the changes in variability metrics, including pupil size, fissure length, blink duration, and blink interval, across the 1800-frame interval during the text-reading task in both groups, corresponding to the three increasing difficulty levels of the reading segments. Statistically significant differences were observed in several parameters. In the nondeteriorated asthenopia group, participants exhibited significantly greater variability in pupil size across the 600-1200–frame interval, as indicated by a higher variance (median 0.64, IQR –0.27 to 1.81, vs median –0.22, IQR –1.25 to 0.68; *P*=.005) and coefficient of variation (median 3.77, IQR –3.78 to 10.48, vs median –3.31, IQR –9.62 to 0.61; *P*=.002). Similarly, fissure length variability was significantly higher in this frame interval in the nondeteriorated asthenopia group, with a higher SD (median 1.22, IQR –0.21 to 4.24, vs median –0.12, IQR –2.01 to 0.58; *P*=.007), variance (median 16.61, IQR –3.31 to 72.81, vs median –1.27, IQR –25.00 to 7.09; *P*=.003), and coefficient of variation (median 3.72, IQR 0.06-10.24, vs median –2.10, IQR –9.09 to 0.23; *P*<.001). In addition, across all frame intervals, the coefficient of variation for fissure length was significantly higher in the nondeteriorated asthenopia group (median –0.88, IQR –7.23 to 0.92, vs median 1.85, IQR –1.71 to 6.27; *P*=.006). No significant differences were found in blink duration or blink interval variability metrics (*P*>.05).

**Table 4:** Comparison of differences in variability metrics of pupil size before and after visual tasks in participants with and without deteriorated asthenopia.

Difference in variability metric and frame interval			With deteriorated asthenopia		Without deteriorated asthenopia	*P* value
**SD, median (IQR)**						
	200-600	0.23 (–0.10 to 0.59)		0.16 (–0.53 to 0.62)		.2
	600-1200	–0.11 (–0.46 to 0.30)		0.34 (–0.25 to 0.64)		.007
	1200-1800	–0.03 (–0.29 to 0.25)		0.06 (–0.23 to 0.31)		.35
	All	–0.01 (–0.17 to 0.19)		0.26 (–0.22 to 0.45)		.16
**Variance, median (IQR)**						
	200-600	0.65 (–0.09 to 1.71)		0.39 (–1.36 to 2.03)		.21
	600-1200	–0.22 (–1.25 to 0.68)		0.64 (–0.27 to 1.81)		.005
	1200-1800	–0.02 (–0.60 to 0.57)		0.11 (–0.73 to 0.66)		.45
	All	–0.02 (–0.20 to 0.45)		0.68 (–0.38 to 1.66)		.11
**Coefficient of variation, median (IQR)**						
	200-600	2.86 (–4.20 to 6.84)		–0.23 (–10.25 to 9.11)		.21
	600-1200	–3.31 (–9.62 to 0.61)		3.77 (–3.78 to 10.48)		.002
	1200-1800	–1.98 (–7.78 to 1.91)		–0.22 (–4.08 to 3.15)		.23
	All	–0.70 (–5.23 to 2.03)		2.18 (–3.22 to 5.59)		.07

**Table 5:** Comparison of differences in variability metrics of fissure length before and after visual tasks in participants with and without deteriorated asthenopia.

Difference in variability metric and frame interval			With deteriorated asthenopia		Without deteriorated asthenopia		*P* value
**SD, median (IQR)**							
	200-600	0.96 (–0.13 to 2.65)		0.66 (–2.17 to 2.17)		.16	
	600-1200	–0.12 (–2.01 to 0.58)		1.22 (–0.21 to 4.24)		.00	
	1200-1800	0.13 (–1.25 to 2.51)		0.43 (–0.78 to 2.28)		.29	
	All	0.11 (–0.48 to 1.30)		1.01 (–0.88 to 2.61)		.28	
**Variance, median (IQR)**							
	200-600	16.10 (–2.18 to 39.78)		9.06 (–25.55 to 27.46)		.17	
	600-1200	–1.27 (–25.00 to 7.09)		16.61 (–3.31 to 72.81)		.003	
	1200-1800	1.57 (–11.38 to 23.01)		7.04 (–8.60 to 20.56)		.43	
	All	1.47 (–5.50 to 14.17)		13.71 (–11.46 to 39.05)		.18	
**Coefficient of variation, median (IQR)**							
	200-600	1.48 (–6.97 to 7.10)		1.84 (–8.67 to 8.17)		.96	
	600-1200	–2.10 (–9.09 to 0.23)		3.72 (0.06 to 10.24)		<.001	
	1200-1800	–2.79 (–9.74 to 2.38)		0.41 (–4.22 to 4.07)		.08	
	All	–0.88 (–7.23 to 0.92)		1.85 (–1.71 to 6.27)		.006	

**Table 6:** Comparison of differences in variability metrics of blink duration and blink interval before and after visual tasks in participants with and without deteriorated asthenopia in all frame intervals.

Parameter and differences in variability metric		With deteriorated asthenopia	Without deteriorated asthenopia	*P* value
**Blink duration**				
	SD, median (IQR)	–12.72 (–16.86 to 2.60)	–1.35 (–63.27 to 10.81)	.51
	Variance, median (IQR)	–876.83 (–36720.3 to 92.59)	–61.73 (–6313.49 to 1000)	.14
	Coefficient of variation, median (IQR)	–11.65 (–70.49 to 0.46)	–5.02 (–34.84 to 8.11)	.26
**Blink interval**				
	SD, median (IQR)	–6.59 (–34.41 to 61.37)	–13.61 (–46.09 to 13.98)	.55
	Variance, median (IQR)	–644.01 (–6785.52 to 13087.04)	–1833.61 (–5870.93 to 2526.15)	.63
	Coefficient of variation, median (IQR)	–2.47 (–10.63 to 6.95)	–2.40 (–14.71 to 16.04]	.96

### Risk Prediction for Deteriorated Asthenopia

**Figure 4:**
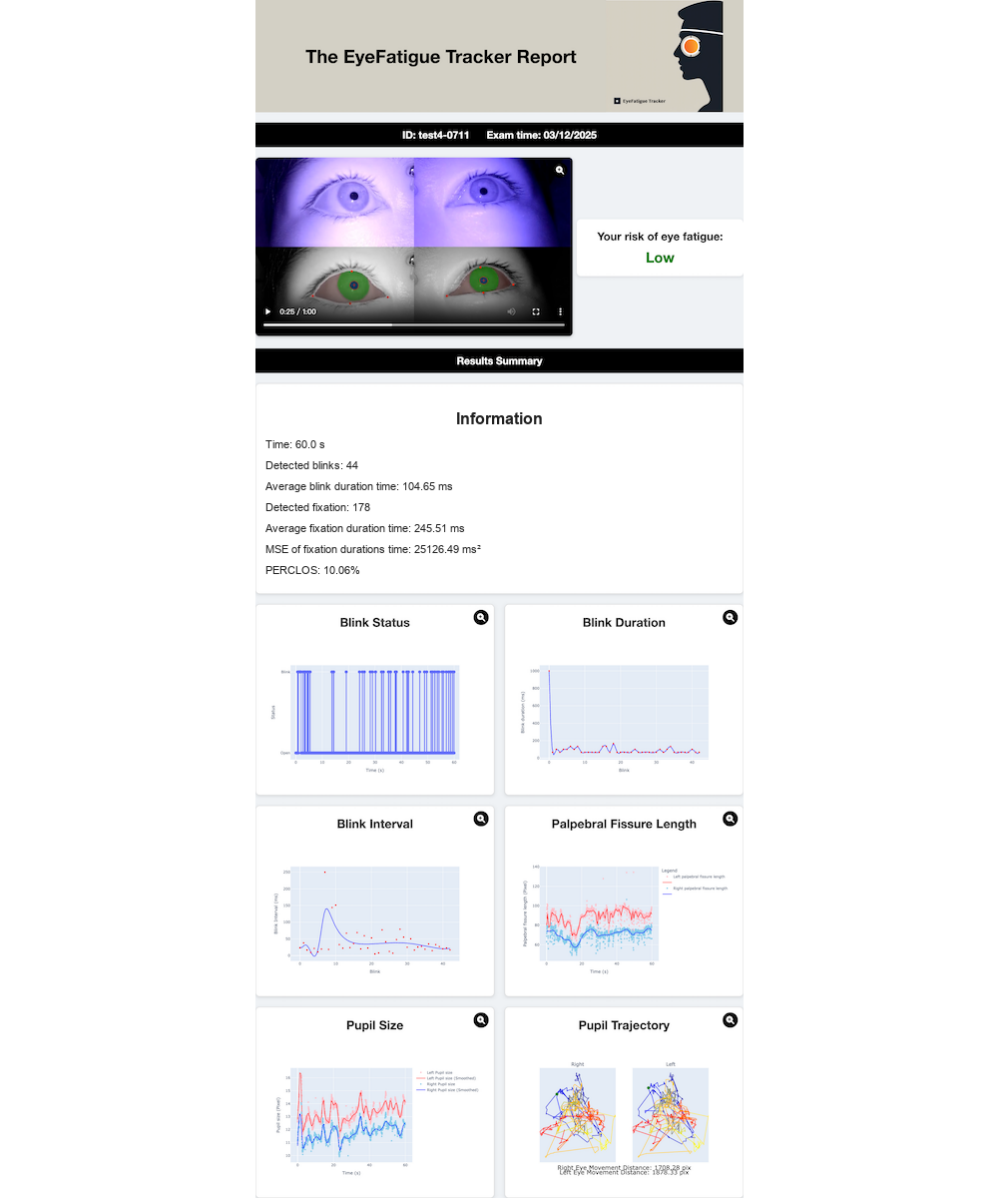
Example report generated by the EyeFatigue Tracker system. The app summarizes changes in ocular parameters during a single 1-minute standardized reading task. The panel on the top shows the IR ocular video with segmentation overlays, and the panel on the right displays the predicted risk level for eye fatigue. The summary block lists key metrics: total time, blink count, mean blink duration, fixation count, mean fixation duration, mean square error of fixation duration, and PERCLOS. The plots depict blink status, blink duration and interval, palpebral fissure length, pupil size, and pupil trajectory for both eyes across the 1-minute task. Values are illustrative. IR: infrared; PERCLOS: percentage of eye closure.

As shown in Table 7, model performance was evaluated using accuracy, AUROC, the *F*_1_-score, and balanced accuracy on the independent test set. Four ML models were built using ten selected features: average blink duration, coefficient of variation of fissure length (600-1200 frames), coefficient of variation of fissure length (200-1800 frames), variance of fissure length (600-1200 frames), SD of fissure length (600-1200 frames), coefficient of variation of pupil size (600-1200 frames), blink times, SD of pupil size (600-1200 frames), variance of pupil size (600-1200 frames), and the CFF. Among the four models, the random forest demonstrated the best overall performance, achieving the highest mean AUROC of 0.850 (95% CI 0.830-0.860), together with the highest accuracy (mean 0.720, SD 0.035), *F*_1_-score (mean 0.771, SD 0.032), and balanced accuracy (mean 0.698, SD 0.042). XGBoost also achieved a high mean AUROC of 0.820 (95% CI 0.800-0.830), whereas the decision tree showed moderate performance across all metrics. In contrast, the SVM exhibited limited discriminative ability, with a balanced accuracy close to the chance level. Table 8 presents the contributions of each feature to the prediction of deteriorated asthenopia using the random forest. The importance scores indicate the relative influence of each feature on the model’s predictive performance, with the average blink duration (score=0.194) and coefficient of variation of fissure length (600-1200 frames, score=0.119) being the most impactful features. Other variability-related features of fissure length and pupil size also contributed nonnegligibly to the model’s predictions, whereas blink times showed a relatively lower contribution. Figure 4 shows the risk report generated by the deployed system. It predicts the deteriorated risk level (high/low), together with the summary metrics and time-series plots of objective metrics. In the prototype, the predicted risk level (low/high) is intended as the main output for lay users, whereas the detailed numerical and time-series indices are primarily provided for clinicians and researchers; future versions will include more user-friendly explanations and simplified summaries of these metrics. Enlarged versions of the individual plots are provided in Figure S3 in Multimedia Appendix 1.

**Table 7:** Prediction performance for detecting deteriorated asthenopia using ML^a^ models.

Model	Accuracy, mean (SD)	*F*_1_-score, mean (SD)	Balanced accuracy, mean (SD)	Mean AUROC^b^ (95% CI)
Decision tree	0.688 (0.046)	0.726 (0.039)	0.682 (0.051)	0.75 (0.73, 0.77)
Random forest	0.720 (0.035)	0.771 (0.032)	0.698 (0.042)	0.85 (0.83, 0.86)
SVM^c^	0.555 (0.039)	0.692 (0.037)	0.511 (0.031)	0.59 (0.56, 0.63)
XGBoost^d^	0.677 (0.048)	0.741 (0.041)	0.661 (0.051)	0.82 (0.80, 0.83)

^a^ML: machine learning.

^b^AUROC: area under the receiver operating characteristic curve.

^c^SVM: support vector machine.

^d^XGBoost: extreme gradient boosting.

**Table 8:** Feature contributions to deteriorated asthenopia prediction in the random forest.

Rank	Feature	Importance score
1	Average blink duration	0.194
2	Coefficient of variation of fissure length (600-1200)	0.119
3	Coefficient of variation of fissure length (200-1800)	0.101
4	Variance of fissure length (600-1200)	0.096
5	SD of fissure length (600-1200)	0.096
6	Coefficient of variation of pupil size (600-1200)	0.087
7	CFF^a^	0.079
8	Variance of pupil size (600-1200)	0.078
9	SD of pupil size (600-1200)	0.077
10	Blink times	0.072

^a^CFF: critical flicker fusion frequency.

## Discussion

### Principal Findings

In this study, we developed and validated EyeFatigue Tracker, an at-home system that classifies the short-term risk for asthenopia deterioration using a standardized 1-minute reading task. The segmentation module performed reliably (overall Dice=93.3% and IoU=87.4%), providing stable masks for downstream analysis. Using these masks, the device objectively quantified ocular metrics related to eye fatigue, including blink patterns, eye movements, and pupil variations. Features reflecting the intratask variability of palpebral fissure length and pupil size, together with blink measures and the CFF, were most informative for prediction. Across the four ML models, the random forest demonstrated the most balanced and robust overall performance on the test split, achieving the highest *F*_1_-score (mean 0.771, SD 0.032), with a mean accuracy of 0.720 (SD 0.035) and a mean AUROC of 0.85 (95% CI 0.830-0.86). Taken together, these results suggest that brief, home-collected ocular signals can support practical monitoring of asthenopia in daily digital use and may offer new insights into objective biomarkers of visual fatigue.

#### Blink Behavior as a Compensatory Mechanism

Previous studies have reported a significant association between blink patterns and asthenopia [47,48]. In this study, the average blink duration and blink times emerged as key predictors of deteriorated asthenopia in the random forest, exhibiting notable differences between the deteriorated and nondeteriorated asthenopia groups. Specifically, the average blink duration increased in the nondeteriorated asthenopia group but remained unchanged in the deteriorated asthenopia group, consistent with prior research suggesting that prolonged blink duration serves as a compensatory mechanism to alleviate visual discomfort [27,49]. This may indicate that individuals experiencing deteriorated asthenopia fail to exhibit an adaptive increase in blink duration, potentially leading to inadequate tear film replenishment and worsened eye strain. Additionally, blink times also contributed to the prediction of deteriorated asthenopia, further supporting the idea that increased blink frequency is often a compensatory response to increasing eye fatigue [50]. These findings indicate that individuals experiencing deteriorated asthenopia may be less able to implement compensatory blink behavior effectively, exacerbating their visual discomfort and strain.

#### Ocular Variability as a Biomarker of Deteriorated Asthenopia

Ocular instability, reflected in fluctuations of fissure length (fissure height) and pupil size, emerged as a key indicator of deteriorated asthenopia. Among all features, the coefficient of variation of fissure length (600-1200 frames) ranked among the most influential predictors (0.119), together with its coefficient of variation in a later interval (200-1800 frames; 0.101), as well as its variance (0.096) and SD (0.096). Similarly, the coefficient of variation of pupil size (600-1200 frames; 0.087) also played a notable role in the prediction model. Notably, variability changes were higher in the nondeteriorated asthenopia group, suggesting an active and functional neuromuscular response to counteract fatigue. In contrast, the deteriorated asthenopia group exhibited lower variability changes, which may indicate fatigue-induced rigidity, where the eyelid and pupillary muscles fail to adjust dynamically, leading to sustained strain and discomfort. This distinction supports the idea that fissure height and pupil size variability reflect an individual’s ability to adapt to visual fatigue.

Previous research suggests that eyelid position influences tear film stability, which may contribute to asthenopia [51]. Additionally, a variability change of fissure length may reflect the orbicularis oculi muscle's response to visual fatigue [52]. Cen et al [53] introduced pupil size as the most significant feature for SVM-based visual comfort prediction. Hopkinson [54] suggested that measuring oscillations in pupillary diameter is one of the simplest methods for assessing visual fatigue. Similarly, Geacintov and Peavler [55] observed that oscillation patterns in pupil size increase with visual fatigue, further supporting its relevance as a fatigue indicator. The variability metrics of pupil size during the specified intervals may reflect underlying oscillatory patterns associated with fatigue, aligning with prior evidence of its sensitivity to fatigue-related changes. These findings suggest that these metrics may therefore serve as potential biomarkers for distinguishing between individuals prone to deteriorated asthenopia [56]. Interestingly, variability metrics within the 600-1200–frame interval were more predictive than those spanning the entire reading task (200-1800 frames). This can be attributed to the task design, which progressively increased in difficulty across three stages. In the first stage (200-600 frames), the task was relatively easy, minimizing visual strain and limiting fluctuations of fissure length and pupil size. In the final stage (1200-1800 frames), the task became highly demanding, potentially triggering compensatory fixation strategies that suppressed variability in both groups. In contrast, the middle stage (600-1200 frames) represented a transition from low-to-moderate difficulty, requiring sustained attention and accommodation. This highlights the 600-1200–frame interval as a critical window where ocular instability becomes most pronounced, making it a sensitive marker for detecting individuals prone to deteriorated asthenopia.

#### Role of CFF and Fixation Parameters

Although no statistically significant difference in the CFF was observed between the deteriorated and nondeteriorated asthenopia groups, its inclusion in the predictive model was based on its recognized role in assessing eye fatigue susceptibility [10]. The CFF has long been established as a psychophysical marker of visual fatigue, as it reflects temporal resolution thresholds in the visual system that deteriorate under asthenopia-inducing conditions [57,58]. Although its individual contribution to the model was lower than most other ocular variability metrics, the CFF remains a valuable input, complementing blink- and pupil-related parameters to improve prediction accuracy.

In contrast, fixation parameters showed limited utility in distinguishing between the two groups in this study. Although Wang et al [13] reported that the average fixation duration increases and fixation numbers decrease as eye fatigue worsens, our findings aligned with their observations in both groups. Nevertheless, these changes were not statistically significant enough to differentiate the deteriorated and nondeteriorated asthenopia groups. One possible explanation is that visual fatigue did not deteriorate considerably during the computer gameplay process, reducing the sensitivity of fixation parameters as discriminative indicators in this context.

### Clinical and Practical Implications of the EyeFatigue Tracker

Building on these observations, our findings contribute to advancing the diagnostics and prediction of deteriorated asthenopia by addressing key limitations in previous studies. Previous research has often relied on head-mounted devices to analyze single-eye metrics, such as pupil accommodation speed [14], blink frequency [15], and eye closure duration [14], or has relied on eye trackers to extract pupil size and eye movement parameters, which are typically unable to directly capture blink pattern data [13]. In contrast, our EyeFatigue Tracker captures binocular ocular videos and a segmentation-driven pipeline to derive an integrated set of metrics for at-home use. Its portability and cost-effectiveness further enhance its potential for pragmatic monitoring in daily digital use. From a health care perspective, this at-home, session-based assessment could help detect early worsening of visual fatigue in high-risk users and provide objective measures to evaluate simple interventions. In telemedicine, the automatically generated reports can be shared with eye care professionals to complement questionnaires and guide follow-up. It may also be applied in clinical settings for the detection and follow-up of asthenopia, providing objective metrics alongside subjective assessments. Moreover, by introducing within-task variability measures as candidate indicators of deterioration, this study suggests potential gains in predictive performance and diagnostic utility.

### Limitations and Future Directions

This study has several limitations that require consideration. First, the relatively small sample size may increase the risk of false-negative results when comparing the objective metrics between the deteriorated and nondeteriorated asthenopia groups and limits the generalizability of our findings. Future studies should recruit larger and more diverse populations to enhance statistical power and broader applicability. In addition, statistical feature filtering (*P*<.05) was used to define candidate ocular biomarkers and was not re-estimated within each cross-validation fold; this design choice may introduce selection bias and lead to optimistic performance estimates. Moreover, performance showed nonnegligible variability across cross-validation folds, underscoring uncertainty under the limited sample size and the need for external validation in independent cohorts. Second, no severe asthenopia cases were observed during the experiment. Including participants with severe symptoms in future studies would provide deeper insights into the relationship between objective metrics and severe visual fatigue. Third, the cohort was studied at a single site under a standardized protocol; subsequent research could incorporate diverse clinical or real-world settings (eg, workplaces with prolonged screen use or clinical contexts involving pre- and postsurgical patients) to better validate the predictive value of objective metrics in broader contexts. Fourth, future technical work could aim to improve the segmentation of challenging structures, such as the palpebral fissure. In addition, although a simple overexposure suppression step improved segmentation stability in our dataset, future versions of the system could integrate more advanced illumination enhancement methods to further increase robustness under challenging at-home lighting conditions. Fifth, we did not systematically test different video frame rates or learning rate schedules; future studies should compare alternative frame rates and apply learning rate–scheduling strategies (eg, step decay or plateau-based reduction) to further optimize the balance between accuracy and computational cost.

### Conclusion

This study developed an at-home system that estimates the short-term risk for asthenopia deterioration after computer use and highlights objective biomarkers that may support earlier detection and prevention. Our findings indicate that brief, home-collected signals can enable practical monitoring and add objective evidence to symptom-based assessment. These conclusions should be interpreted in the light of the methodological and study limitations, including the modest sample size, the single-site design, and the use of short-term changes in CVS-Q scores as the reference outcome. Future work should validate generalizability in larger and more diverse cohorts, assess longitudinal stability in routine use, and evaluate performance in clinically severe cases.

## Appendix

Multimedia Appendix 1 Data collection workflow and representative task materials, additional segmentation examples under different eye-closure conditions, enlarged views of the six analytic panels in the EyeFatigue Tracker risk report, parameter search grid, and comparison of subjective metrics before and after the visual tasks.

Multimedia Appendix 2 Standardized 1-minute text-reading task materials used before and after the game sessions.

## Abbreviations

AP: average precision
AR: average recall
AUROC: area under the receiver operating characteristic curve
CFF: critical flicker fusion frequency
COCO: Common Objects in Context
CVS-Q: Computer Vision Syndrome Questionnaire
Dice: Dice similarity coefficient
DL: deep learning
FAS: Fatigue Assessment Scale
fps: frames per second
GPU: graphics processing unit
HVA: habitual visual acuity
IoU: intersection over union
IR: infrared
KSS: Karolinska Sleepiness Scale
mark R-CNN: mask region-based convolutional neural network
ML: machine learning
PERCLOS: percentage of eye closure
SSS: Stanford Sleepiness Scale
SVM: support vector machine
TBUT: tear film break-up time
XGBoost: extreme gradient boosting
