# Supplementary material for: Identifying Biomarkers Using a Portable, Home-Based Eye-Tracking System to Predict Short-Term Visual Fatigue Deterioration: Prospective Observational Feasibility Study
Source: JMIR Hum Factors. 2026 Apr 28;13:e84479. doi: 10.2196/84479 (PMC13168858; doi:10.2196/84479)
Supplement: Multimedia Appendix 1 [file humanfactors_v13i1e84479_app1.docx]

Multimedia Appendix 1. Supplementary figures and tables.

Figure S1. Data collection workflow and representative task materials.

Figure S2. Additional segmentation examples under different eye-closure conditions.

Figure S3. Enlarged views of the six analytic panels in the EyeFatigue Tracker risk report.

Table S1. Parameter search grid.

Table S2.Comparison of subjective metrics before and after the visual tasks.

Figure S1. Data collection workflow and representative task materials. Participants completed pre-task questionnaires (CVS-Q, SSS, FAS, KSS), HVA assessment, TBUT measurement, and CFF testing, followed by a 1-minute EyeFatigue Tracker assessment during a standardized text-reading task (eye videos recorded). They then performed a 1-hour visual task consisting of four consecutive 15-minute web-based mini-games: (A) maze navigation (guiding an on-screen cursor to the goal), (B) block-stacking (Tetris; aligning and stacking falling blocks), (C) obstacle avoidance (jumping upward to avoid on-screen obstacles), and (D) a sports-themed mini-game (search-and-find task requiring visual scanning) [1-4]. Post-task assessments repeated the same questionnaires, HVA assessment, TBUT measurement, CFF testing, and the 1-minute EyeFatigue Tracker reading assessment. The EyeFatigue Tracker was worn only during the 1-minute pre- and post-task reading assessments (not during the 1-hour gameplay). HVA, habitual visual acuity (HVA); TBUT, tear film break-up time; CVS-Q, Computer Vision Syndrome Questionnaire; SSS, Stanford Sleepiness Scale; KSS, Karolinska Sleepiness Scale; FAS, Fatigue Assessment Scale; CFF, critical flicker fusion frequency.

**
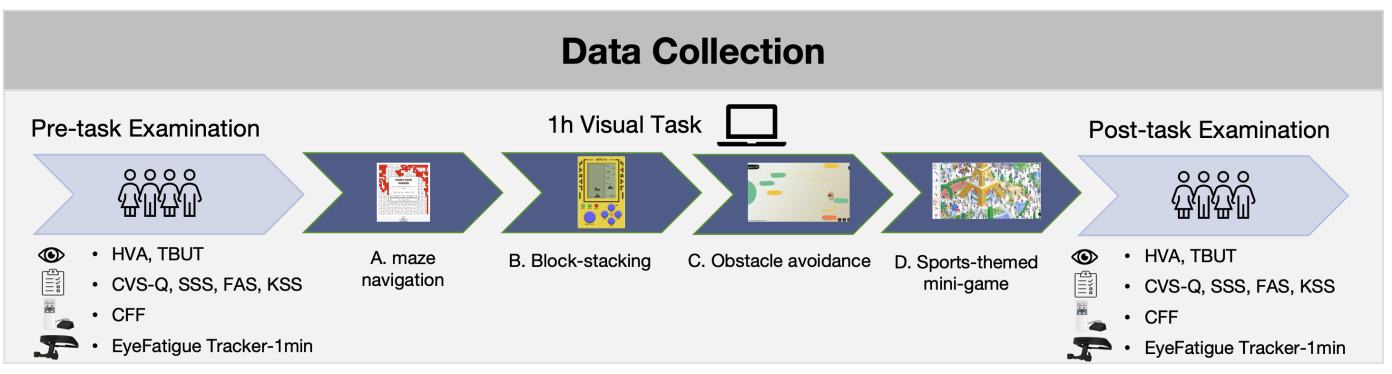
**

Figure S2. Additional segmentation examples under different eye-closure conditions. (A) Fully open eyes; (B) partially open eyes in both eyes; (C) asymmetric partial closure, where one eye remains partially open and the pupil and iris are still segmented, while the fellow eye is almost closed and therefore shows no pupil or iris segmentation; (D) fully closed eyes, where no pupil or iris segmentation is produced.


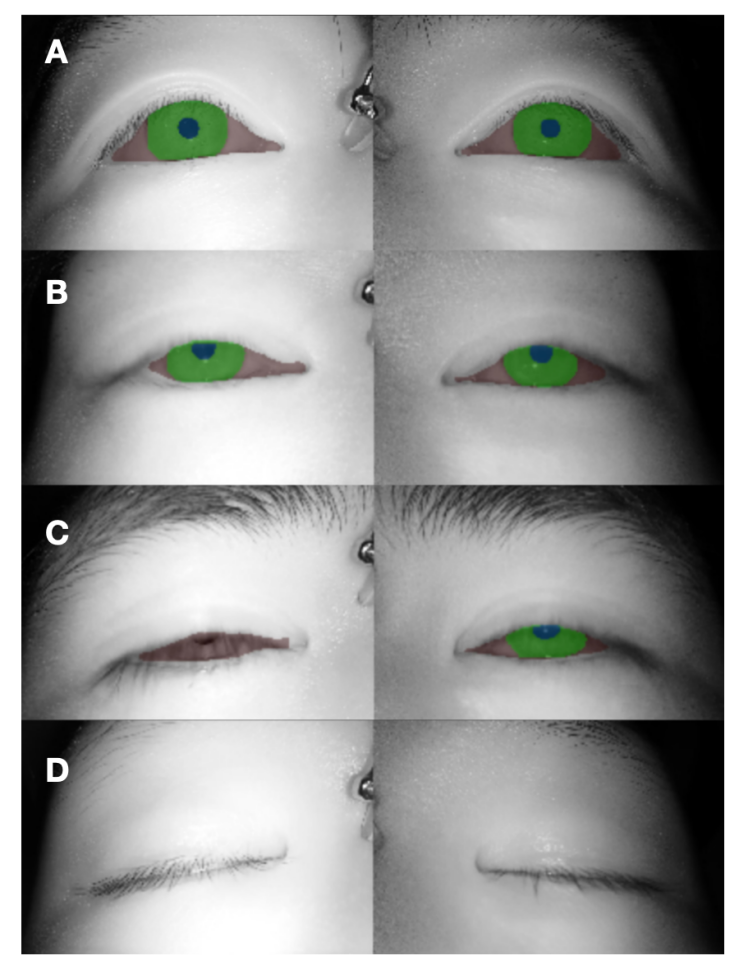


Figure S3. Enlarged views of the six analytic panels in the EyeFatigue Tracker risk report. (A) Blink status; (B) Blink duration; (C) Blink interval; (D) Palpebral fissure length; (E) Pupil size; and (F) Pupil trajectory. pix denotes pixels. Time is shown in seconds (s) and blink-related measures in milliseconds (ms).


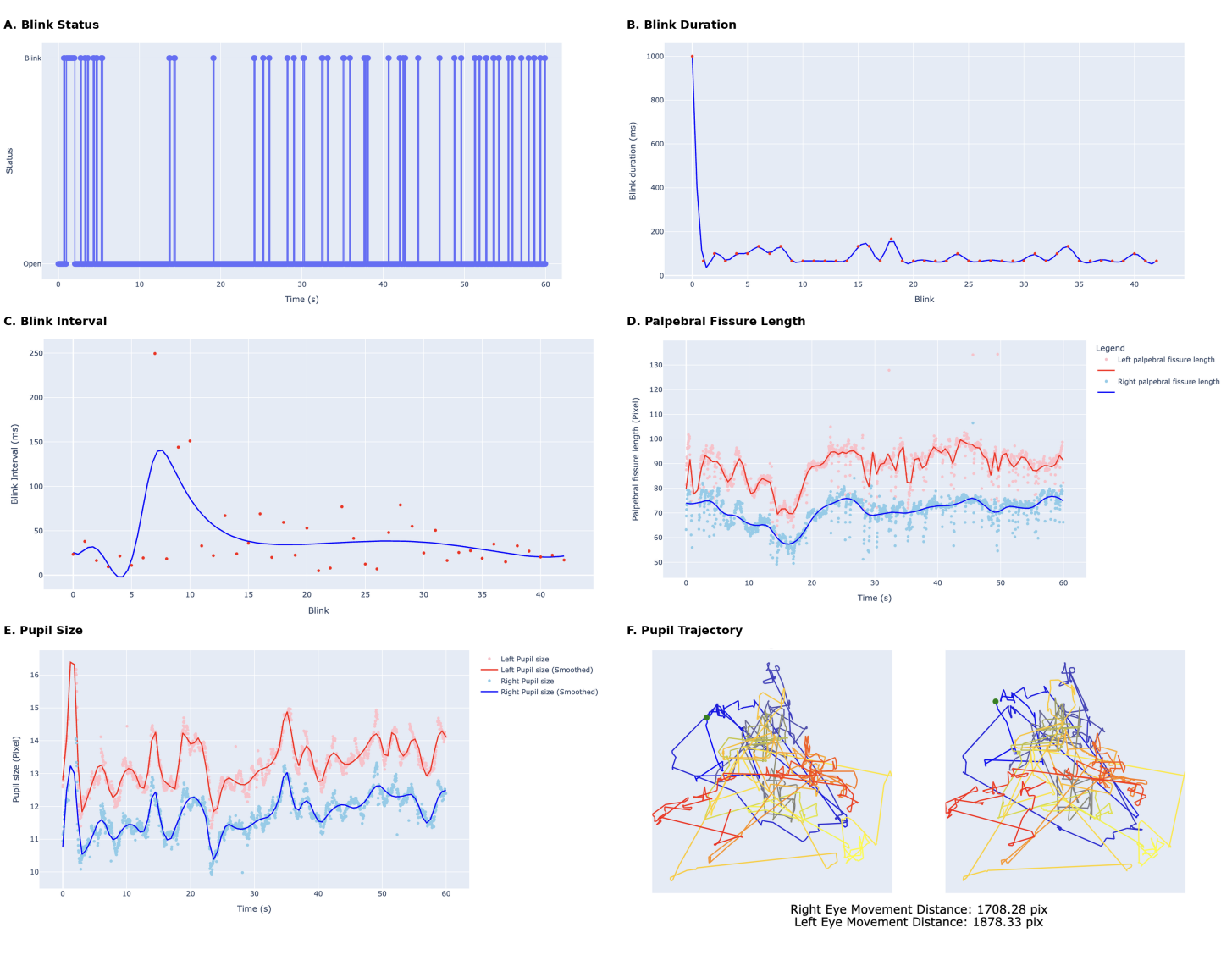


Table S1. Parameter search grid. SVM, support vector machine; XGBoost, extreme gradient boosting.

| **Model** | **Parameter Grid** |
| --- | --- |
| SVM | kernel ∈ {rbf, poly, sigmoid}  C ∈ {0.1, 1, 10}  gamma ∈ {scale, auto, 0.1}  class_weight ∈ {balanced, None}  shrinking ∈ {True}  tol ∈ {1e−3, 1e−4}  degree ∈ {2, 3} (for polynomial kernel)  coef0 ∈ {0.0, 0.5} (for polynomial and sigmoid kernels) |
| **Decision Tree** | max_depth ∈ {2, 3, 4, 5}  min_samples_split ∈ {2, 4, 6, 8}  min_samples_leaf ∈ {1, 2, 4, 6}  criterion ∈ {gini, entropy}  class_weight ∈ {balanced}  ccp_alpha ∈ {0.0, 0.001, 0.005, 0.01} |
| **Random Forest** | n_estimators ∈ {200, 400}  max_depth ∈ {None, 5, 10}  min_samples_split ∈ {2, 5, 10}  min_samples_leaf ∈ {1, 2, 4, 8}  max_features ∈ {sqrt, 0.5}  class_weight ∈ {None, balanced}  bootstrap ∈ {True} |
| **XGBoost** | n_estimators ∈ {100, 300}  max_depth ∈ {2, 3, 4}  learning_rate ∈ {0.03, 0.1}  subsample ∈ {0.6, 0.8}  colsample_bytree ∈ {0.6, 0.8}  gamma ∈ {0, 0.1}  reg_alpha ∈ {0.0, 0.5, 1.0}  reg_lambda ∈ {1.0, 5.0}  min_child_weight ∈ {1, 5, 10} |

Table S2 presents the results of four subjective questionnaires, alongside the major complaints related to asthenopia, before and after the visual tasks. All subjective questionnaire metrics showed significant deterioration following the visual tasks. Specifically, participants’ CVS-Q scores were higher after visual tasks compared to baseline values (mean [SD], 9.21 [4.57] vs. 6.76 [3.76], P < .001). The prevalence of participants in the higher severity group (scores of 13-18) increased from 3 of 38 (8%) at baseline to 13 of 38 (34%), while those classified as without asthenopia (scores of < 6) decreased from 16 of 38 (42%) to 12 of 38 (32%). Additionally, levels of SSS, KSS, and FAS all slightly increased post-visual tasks, with median [IQR], 1 [0-2], 1 [0-2], 1 [0-4], respectively. Notably, the number of participants reporting the most common complaint-dryness rose by 4 [-6%, 27%], while those reporting excessive blinking saw a substantial increase of 15 [21%, 58%]. Together, these results show that the task led to more pronounced short-term worsening of visual fatigue than of general tiredness. In the following analyses, we therefore focus on how changes in ocular metrics are related to CVS-Q–defined deterioration in asthenopia.

Table S2. Comparison of subjective metrics before and after the visual tasks. Abbreviations: SD, standard deviation; IQR, interquartile range; n, number; CVS-Q, Computer Vision Syndrome Questionnaire; SSS, Stanford Sleepiness Scale; KSS, Karolinska Sleepiness Scale; FAS, Fatigue Assessment Scale.

| **Parameter** | **Before visual tasks** | **After visual tasks** | **Difference**  **[95%CI]** | ***P* value** |
| --- | --- | --- | --- | --- |
| CVS-Q Scores |  |  |  |  |
| Mean ± SD | 6.76 ± 3.76 | 9.21 ± 4.57 | 2.45 [1.60, 3.29] | < .001 |
| CVS-Q Severity |  |  |  |  |
| <6 [n, %] | 16 [42%] | 12 [32%] |  | < .001 |
| 6-12 [n, %] | 19 [50%] | 13 [34%] |  |  |
| 13-18 [n, %] | 3 [8%] | 13 [34%] |  |  |
| SSS level, median [IQR] | 3 [2-3] | 4 [3-5] | 1 [0-2] | < .001 |
| KSS level, median [IQR] | 4.50 [3-6] | 6 [4-7] | 1 [0-2] | .001 |
| FAS level, median [IQR] | 18.50 [15-25] | 23 [20-28] | 1 [0-4] | < .001 |
| Complaint of Dryness, n [%] | 27 [71%] | 31 [82%] | 4 [-6 %, 27%] | .16 |
| Complaint of Excessive blinking, n [%] | 18 [47%] | 33 [87%] | 15 [21%, 58%] | < .001 |

## References

1. Maze Toys. Mighty Maze. Accessed on August 1, 2024. https://maze.toys/mazes/mighty/
2. Chvin. React Tetris. Accessed on August 1, 2024. https://chvin.github.io/react-tetris/
3. 12wave. Game Over. Accessed on August 1, 2024. https://12wave.com/gameover
4. Google. Search Playground: Paris 2024. Accessed on August 1, 2024. https://searchplayground.google/intl/en/paris-2024/
